# Kidney Transplant Outcomes in Recurrent Versus *De Novo* IgA Nephropathy

**DOI:** 10.1016/j.ekir.2025.05.014

**Published:** 2025-05-21

**Authors:** Mark Haas, James Mirocha, Hae Yoon Grace Choung, Jean Hou, Mercury Y. Lin, Michifumi Yamashita, Cynthia C. Nast

**Affiliations:** 1 Department of Pathology and Laboratory Medicine, Cedars-Sinai Medical Center, Los Angeles, California, USA; 2 General Clinical Research Center, Cedars-Sinai Medical Center, Clinical & Translational Science Institute, Los Angeles, California, USA

**Keywords:** complement, IgA nephropathy, kidney transplant, Oxford classification, recurrent glomerulonephritis

## Abstract

**Introduction:**

IgA nephropathy (IgAN) recurs frequently in kidney transplants, although the reported frequency of recurrence varies because many studies do not distinguish recurrent IgAN from IgAN occurring *de novo* in the allograft. In addition, there are only limited studies of graft outcomes in patients with recurrent IgAN.

**Methods:**

We retrospectively examined 264 kidney transplant biopsies with glomerular IgA deposits, distinguishing whenever possible recurrent versus *de novo* IgAN, and compared clinical and pathologic features of biopsies of patients within these groups. We also examined graft outcomes in patients with available follow-up to identify findings associated with graft survival.

**Results:**

A significantly greater fraction of biopsies with recurrent disease (*n* = 127) had mesangial (M1) and endocapillary (E1) hypercellularity, crescents (C1-2), and > 1+ (0–4+ scale) glomerular C3 staining than biopsies with *de novo* (*n* = 46) IgAN; there was no significant difference between these groups with respect to Oxford S and T scores, or mean time posttransplantation of the biopsy. None of 19 biopsies with IgA deposits attributable to the donor had any MEST-C scores > 0 or C3 staining > 1+. Of 54 patients with recurrent IgAN and follow-up, 25 lost their grafts between 2 and 109 months postbiopsy. Findings at the time of biopsy significantly associated with an increased risk of graft loss in patients were higher serum creatinine (SCr) and proteinuria; Oxford E, T, and C scores > 0; and C3 > 1+.

**Conclusion:**

The findings indicate the importance of measuring proteinuria in patients with suspected recurrent IgAN and scoring such biopsies using the Oxford MEST-C scores.

## Graphical abstract

**Figure:**
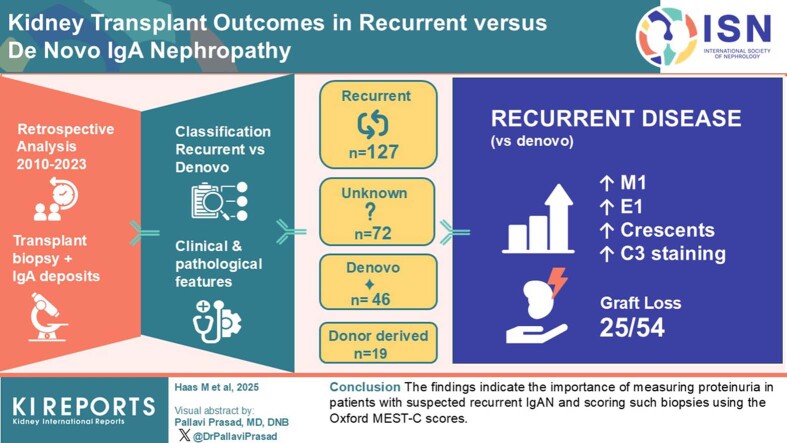

See Commentary on Page 2539

IgAN is the most common form of primary glomerulonephritis in the world,^1^ and recent studies have shown that although its progression tends to be relatively slow, it eventually leads to the development of end-stage kidney disease requiring dialysis or transplantation in > 50% of adult patients and > 30% of pediatric patients by 2 decades after diagnosis.^2^ IgAN also recurs frequently in kidney transplants, although the reported frequency of recurrence varies significantly because the criteria used to define such recurrences vary greatly.^3^ In addition, studies of IgAN in kidney allografts often do not distinguish recurrent IgAN from IgAN occurring *de novo* in the transplanted kidney.^4^, ^5^, ^6^, ^7^, ^8^ There are also no uniform criteria for evaluating the histologic and clinical severity of recurrent IgAN or for determining when and how to treat such recurrences, although a recent multicenter study showed that graft survival in patients with recurrent IgAN is negatively impacted by proteinuria at the time of recurrence and to a lesser extent by an associated rise in SCr.^9^

In addition, there have been few studies examining the association of pathologic findings, including the Oxford MEST-C scores, on graft outcomes in patients with recurrent IgAN, although several studies have noted a negative association between crescents and graft survival.^7^^,^^8^^,^^10^, ^11^, ^12^ Two Korean studies^6^^,^^8^ found an association between endocapillary hypercellularity (Oxford E1 score), segmental glomerulosclerosis (S1), and interstitial fibrosis/tubular atrophy (T1-2) and decreased graft survival in patients with IgAN in their kidney allografts, although these studies did not specifically distinguish between recurrent and *de novo* IgAN. Park and coworkers^8^ also found a progressive decrease in the likelihood of allograft survival with an increasing number of MEST-C scores > 0; and a multicenter study of recurrent IgAN by the Banff Recurrent Glomerulonephritis Working Group^12^ found a significant association between the sum of the MEST-C scores and the likelihood of graft loss, in addition to an association with individual E, T, and C scores. However, because non-zero MEST-C scores are also associated with higher levels of SCr and of proteinuria at the time of biopsy,^6^ it remains unclear if the impact of these histologic findings are independent of these clinical parameters, particularly proteinuria which is so strongly associated with worse native kidney outcomes in IgAN^2^ and is currently a key marker used in evaluation of drug efficacy in treating native kidney IgAN.^13^

In this single-center study, we retrospectively examined 264 kidney transplant biopsies with glomerular IgA deposits performed over a 14-year interval, distinguishing whenever possible recurrent versus *de novo* IgAN, comparing clinical and pathologic features of biopsies of patients within these groups. Biopsy findings examined included not only MEST-C scores but the intensity of glomerular staining for C3, a component of the alternative pathway of complement, which in native kidney IgAN has been found to be associated with clinical outcomes and represents a potential therapeutic target.^14^, ^15^, ^16^, ^17^ We also examined graft outcomes in patients with available follow-up to identify findings associated with graft survival.

## Methods

### Patients and Biopsies

Computerized records of the Department of Pathology and Laboratory Medicine, Cedars-Sinai Medical Center, were searched to identify all kidney transplant biopsies performed from January, 2010 to December, 2023 showing glomerular immunofluorescence staining for IgA with an intensity of ≥ 1+ on a scale of 0 to 4+; biopsies with trace or trace to 1+ staining were not included. Our center routinely performs direct immunofluorescence studies for IgG, IgA, IgM, C1q, C3, and kappa and lambda light chains on kidney transplant biopsies other than zero-time (postreperfusion) biopsies. Other exclusion criteria included a diagnosis other than IgAN, such as lupus nephritis, IgA dominant postinfectious glomerulonephritis, IgA deposition secondary to liver disease, or IgA vasculitis; repeat biopsies from the same patient after an initial transplant biopsy with IgA deposits, and biopsies diagnostic for active or chronic active antibody-mediated rejection (AMR)^18^; the latter were excluded because such biopsies typically show glomerulitis that cannot be distinguished from endocapillary hypercellularity and thus preclude accurate scoring of the Oxford E lesion. For each biopsy meeting these criteria, the following pathologic findings were recorded from the pathology report: Oxford scores^19^, ^20^, ^21^ for mesangial hypercellularity (M0 or M1), endocapillary hypercellularity (E0 or E1), segmental glomerulosclerosis (S0 or S1), interstitial fibrosis/tubular atrophy (T0, T1, or T2), and crescents (C0, C1, or C2; for biopsies done before 2017, this score was determined from the fraction of nonglobally sclerotic glomeruli with cellular and/or fibrocellular crescents as enumerated in the microscopic description), as well as the intensity of glomerular C3 staining and presence or absence of cell-mediated rejection (CMR) (Banff grade 1A or greater). Since 2010, all biopsies read at our center with glomerular IgA deposits were scored using the MEST or MEST-C scores; in the small number of such cases where the report did not contain such scores the slides were reviewed by 1 pathologist (MH) and the scores recorded. Other information recorded at this time included the patient’s native kidney disease, patient’s age at the time of biopsy, patient’s sex, time posttransplantation of biopsy, biopsy date, surgical pathology and patient record numbers. The latter number was then used to search Cedars-Sinai computerized records (Cedars-Sinai Link) for the following information: donor type (deceased, living related, or living unrelated), SCr level and urine protein-to-creatinine ratio (Up/c) at the time of biopsy, and whether the patient developed graft loss with return to dialysis (if so, the time postbiopsy of graft loss and if not the time between the biopsy and last follow-up or death with a functioning graft). All biopsy and clinical information were recorded using study numbers (1–264); after all data were recorded links of study numbers to patient identifiers were destroyed. Follow-up information was only available for those patients followed-up with at Cedars-Sinai; the majority of our kidney biopsies are received from over 30 outside hospitals and laboratories in California and 4 other states. Therefore, analysis of clinical outcomes was limited to 74 patients, 54 with recurrent IgAN and 20 with *de novo* IgAN.

### Statistical Analysis of Data

Morphological and clinical parameters were expressed as means and SD for normally distributed variables and as medians and interquartile ranges for nonnormally distributed variables. Differences in development of graft loss between patient groups were analyzed using the Kaplan-Meier method with log-rank tests to determine significance, and Cox proportional hazards models to determine hazard ratios and their 95% confidence intervals. All tests were 2-tailed, and significance was defined as *P* < 0.05. SAS version 9.4 (SAS Institute, Cary, NC) was used for calculations.

## Results

### Biopsy Findings

From 2010 to 2023, a total of 264 kidney transplant biopsies (4.3%) processed at our center showed ≥ 1+ glomerular IgA staining by immunofluorescence and met entry criteria for this study. This included 192 for which the patient's native kidney disease was documented in the clinical record. All these biopsies were indication biopsies, performed for the following indications: acute or persistent graft dysfunction (*n* = 196), proteinuria (*n* = 52), delayed graft function (*n* = 14), or *de novo* donor-specific antibodies (*n* = 2). Of the 264 total biopsies, 127 were in patients with documented IgAN in their native kidney and 65 were in patients with other diseases as the cause of their native kidney failure. Of the latter 65 patients, 19 (including the 14 biopsied for delayed graft function) had biopsies showing glomerular IgA staining during the first 6 weeks posttransplantation that was felt to be donor-derived, whereas the other 46 were categorized as having *de novo* IgAN. In 72 cases, the cause of native kidney failure was not listed or not known.

In Table 1, we compared pathologic features of biopsies showing recurrent and *de novo* IgAN, as well as those with donor-derived IgA deposits. Notably, of the 19 biopsies in the latter category, none showed any MEST-C score > 0 and none showed > 1+ glomerular staining for C3 by immunofluorescence. Four of these biopsies showed acute CMR. Compared with biopsies with *de novo* IgAN, those with recurrent disease were significantly more likely to have evidence of active glomerular lesions, including mesangial (M1) and endocapillary (E1) hypercellularity, crescents (C1 in all but 2 cases), and C3 > 1+; 8 biopsies with crescents (all with recurrent IgAN) also showed glomerular necrotizing lesions. By contrast, there were no significant differences between these groups with respect to the chronic lesions of segmental glomerulosclerosis (S1) and interstitial fibrosis/tubular atrophy (T1 and 2), perhaps reflecting the similar mean time posttransplantation of biopsies in these groups (Table 1). A greater fraction of biopsies with recurrent disease were done for proteinuria, whereas a smaller fraction showed CMR. The group of 72 biopsies done in patients with unknown cause of native kidney failure tended to have histologic scores for active lesions intermediate between the recurrent and *de novo* groups, although the unknown group had the highest fraction of biopsies with CMR.

**Table 1:** Pathologic and demographic findings in different biopsy categories

Parameter	Recurrent (127)	Unknown (72)	*De novo* (46)	Donor (19)	*P* rec vs. unknown^a^	*P* rec vs. *de novo*^a^
M1	57/127 (45%)	15/72 (21%)	8/46 (17%)	0/19 (0%)	0.0007	0.0001
E1	35/127 (28%)	6/72 (8%)	2/46 (4%)	0/19 (0%)	<0.0001	0.0006
S1	71/127 (56%)	29/72 (40%)	21/46 (46%)	0/19 (0%)	0.039	0.30
T1-2	65/127 (51%)	27/72 (38%)	19/46 (41%)	0/19 (0%)	0.076	0.30
C1-2	20/127 (16%)	4/72 (6%)	1/46 (2%)	0/19 (0%)	0.041	0.016
C3 >1+	75/127 (59%)	20/72 (27%)	7/46 (15%)	0/19 (0%)	<0.0001	<0.0001
Rejection	24/127 (19%)	32/72 (44%)	16/46 (35%)	4/19 (21%)	0.0003	0.0040
Biopsy for proteinuria	36/127 (28%)	10/72 (14%)	6/46 (13%)	0/19 (0%)	0.023	0.045
Patient age (yrs)	47.1 ± 12.2	46.0 ± 13.2	45.1 ± 15.8	43.5 ± 13.3		
(range)	16–78	22–69	17–79	25–68		
Patient sex (M/F)	82/45	48/24	30/16	15/4		
Mo posttransplant	91.1 ± 62.6	85.1 ± 72.5	92.7 ± 71.7			
(range)	3–264	2–319	4–312	0–1.5		

a *P*-values by Fisher exact test. Data for patient age (at the time of biopsy) and months posttransplantation are means ± SD. Differences between donor IgA and the other 3 categories were not tested statistically. Rejection indicates the presence of cell-mediated rejection (Banff grade 1A or greater) on the same biopsy showing glomerular IgA deposits. Of the biopsies with T1–2, 106 had T1 and 5 had T2; all but 2 of the biopsies with crescents had C1. Eight biopsies with crescents also showed segmental necrosis in ≥ 1 glomeruli; all of these patients had recurrent disease.

Not surprisingly, biopsies with M1, E1, as well as C1–2 lesions were significantly more likely to show > 1+ glomerular staining for C3 than those without these lesions, consistent with studies in native kidney IgAN,^15^^,^^16^ although unlike the latter studies we did not find an association between C3 staining and the T lesion (Table 2).

**Table 2:** Association of glomerular C3 deposition with MEST-C scores

Biopsy subset	Fraction (%) with C3 > 1+	*P* value^a^
All biopsies	102/245 (42%)	
M0	50/175 (29%)	<0.0001
M1	52/70 (74%)	
E0	70/205 (34%)	< 0.0001
E1	32/40 (80%)	
S0	44/124 (35%)	0.053
S1	58/121 (48%)	
T0	54/134 (40%)	0.70
T1-2	48/111 (43%)	
C0	80/220 (36%)	< 0.0001
C1-2	22/25 (88%)	

Biopsies with donor-derived IgA deposits are not included. Of the 72 biopsies with cell-mediated rejection 29 of 72 (40%) had C3 >1+; of those performed for proteinuria 24 of 52 (46%) had C3>1+.

a By Fisher exact test.

### Findings Associated With Kidney Outcomes

**Figure 1:**
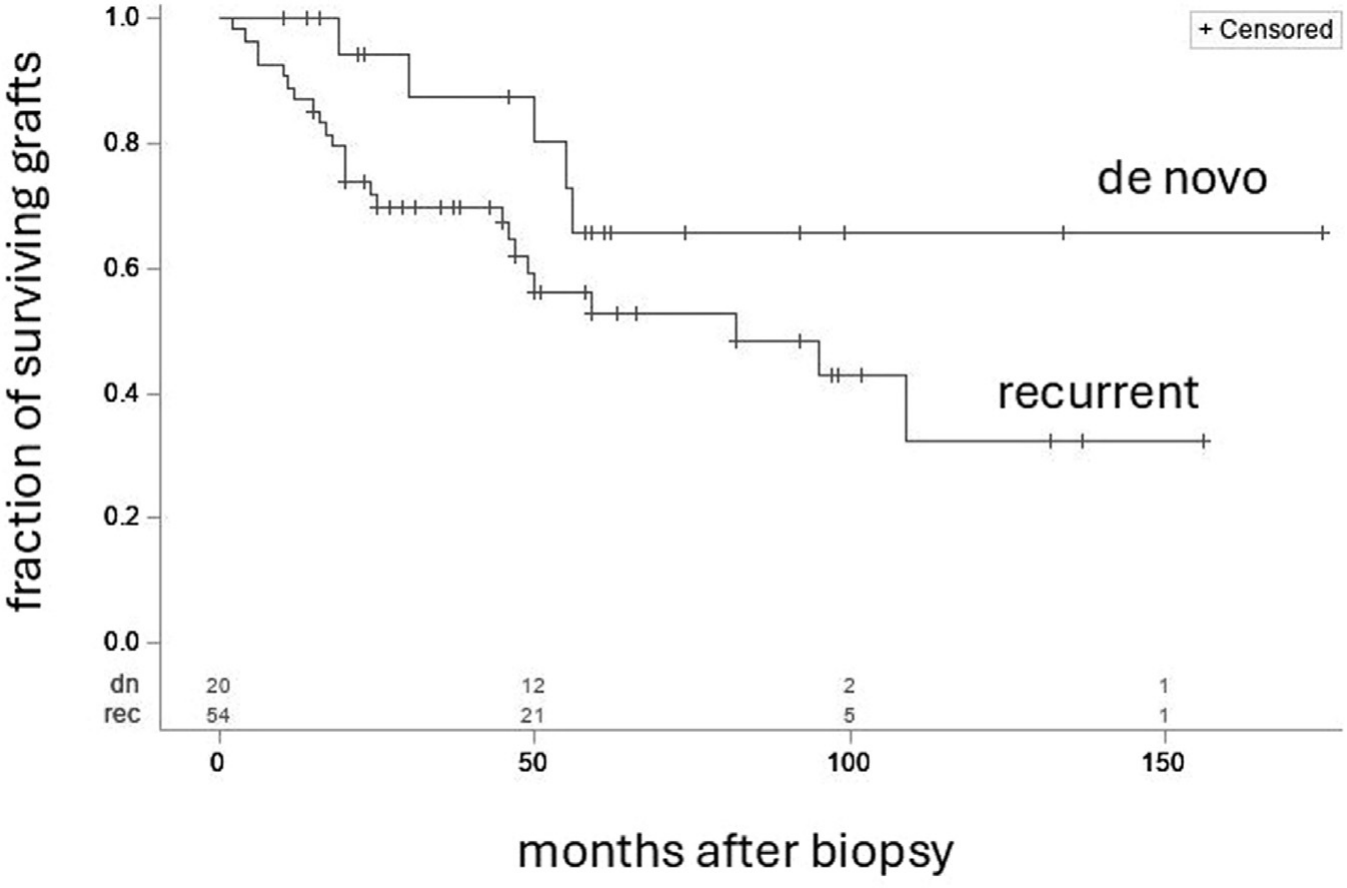
Kaplan–Meier analysis of death-censored graft survival in patients with de novo (*n* = 20) and recurrent (*n* = 54) IgAN. Vertical ticks on each curve indicate censored values (time posttransplantation of last follow-up without graft loss), and the numbers of remaining at risk patients are indicated just above the x-axis. The 2 curves are not significantly different by log-rank (*P* = 0.106) and Wilcoxon analysis (*P* = 0.072). IgAN, IgA nephropathy.

Follow-up data were available for 74 patients, 54 with recurrent IgAN and 20 with *de novo* IgAN; features of these patients are listed in Supplementary Table S1 and are similar to those in the full cohort. Graft survival was worse in patients with recurrent IgAN than in those with *de novo* IgAN (Figure 1), although this did not reach statistical significance (*P* = 0.106 by log-rank test and 0.072 by Wilcoxon, the latter giving somewhat greater emphasis to the earlier follow-up period).

**Figure 2:**
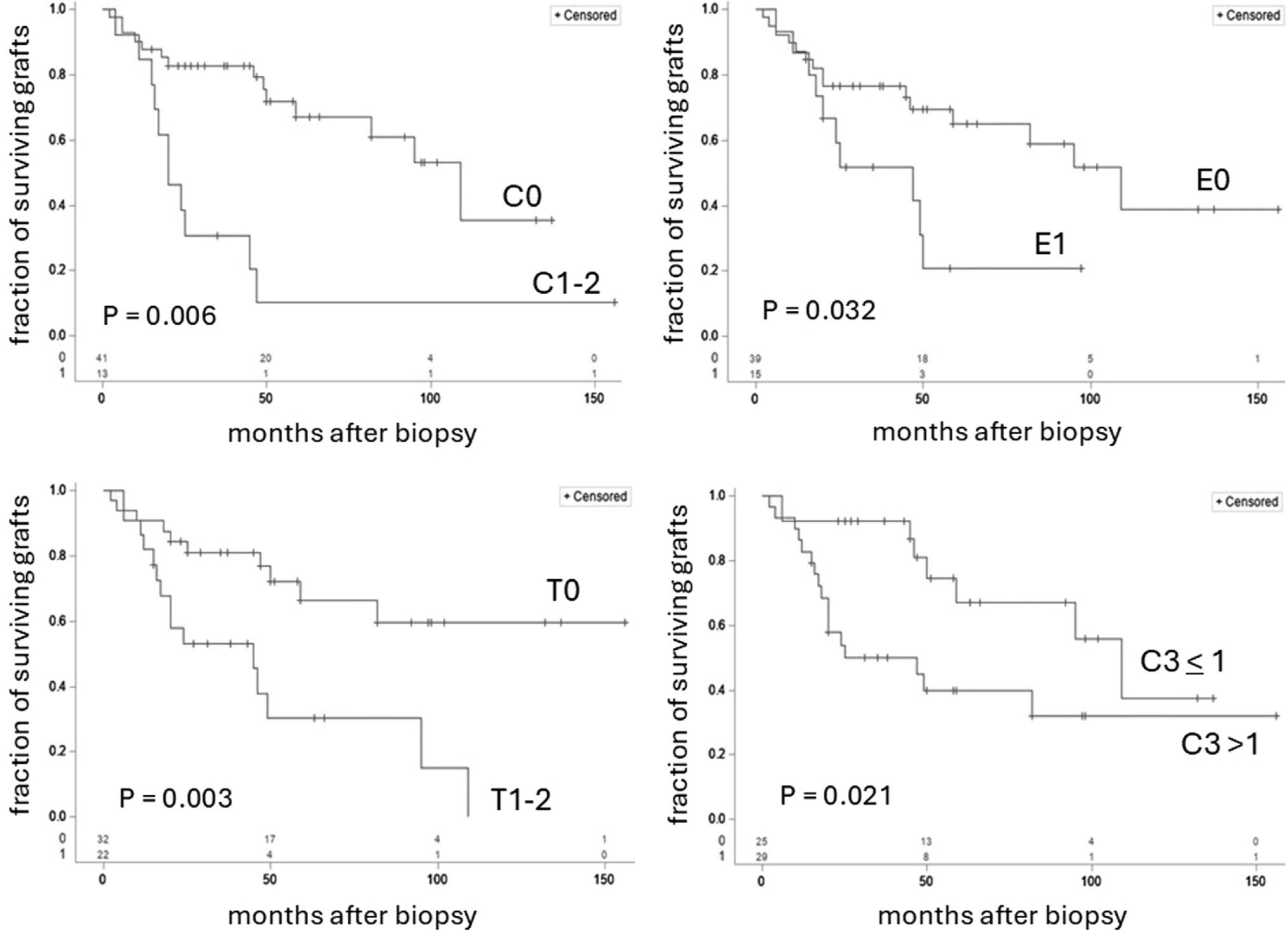
Kaplan–Meier analysis of death-censored graft survival in patients with recurrent IgAN as a function of crescents (C0 vs. C1-2), endocapillary hypercellularity (E0 vs. E1), interstitial fibrosis/tubular atrophy (T0 vs. T1-2), and intensity of glomerular C3 staining (≤1 vs. >1 on a 0 – 4+ scale). Vertical ticks on each curve indicate censored values (time posttransplantation of last follow-up without graft loss), and the numbers of remaining at risk patients are indicated just above the x-axis. In each case the curves are significantly different by both log-rank analysis and Cox analysis; *P*-values for the latter are indicated on each curve. IgAN, IgA nephropathy.

We analyzed histological and clinical findings associated with postbiopsy graft survival in the cohort of 54 patients with recurrent IgAN. By univariate analysis, findings associated with an increased likelihood of graft loss included higher SCr level and Up/c at the time of biopsy; the histologic scores E1, T1–2, and C1–2; and >1+ glomerular C3 staining (Table 3 and Figure 2). Notably, each of these pathologic findings except for E1 were significantly associated with reduced graft survival independent of SCr and Up/c levels; the latter 2 variables remained independent predictors of graft survival rates in all of these in multivariable analyses (Table 3). Concurrent CMR (Banff grade 1A or greater) was associated with a higher risk of graft loss by univariate analysis, although this was not statistically significant, possibly related to additional immunosuppression given to these patients. In a multivariable analysis of T1–2, C1–2, and > 1+ glomerular C3 staining, the former 2 variables were independent predictors of graft loss with hazard ratios of 3.18 and 3.70 and *P*-values of 0.0103 and 0.0012, respectively, whereas C3 staining did not quite reach statistical significance (*P* = 0.0585) despite a robust hazard ratio of 2.32. Interestingly, whereas patients with deceased-donor and living related grafts did not show significantly different graft survival rates by univariate analysis, patients with deceased-donor grafts showed better postbiopsy graft survival in the analysis that also included SCr and Up/c at the time of biopsy. The findings for recurrent IgAN shown in Table 3 are quite similar when considering both recurrent and *de novo* IgAN together (Supplementary Table S2) suggesting the clinical and pathologic risk factors for graft loss appear to be similar with recurrent and *de novo* disease and that differences in outcomes between these groups (Figure 1) are likely to be related in large part to differences in clinical and pathologic severity at the time of biopsy (Table 1 and Supplementary Table S1).

**Table 3:** Clinical and pathologic parameters associated with graft loss in recurrent IgA nephropathy

	Univariate			With SCr and Up/c		
Parameter	Hazard ratio^a^	95% CI	*P*-value	Hazard Ratio^a^	95% CI	*P*-value
Serum creatinine (SCr, mg/dl)	1.87^b^	1.42–2.44	<0.0001			
Urine protein-to-creatinine ratio (Up/c, g/g)	1.43^b^	1.21–1.69	<0.0001			
M1 (vs. M0)	1.63	0.73–3.62	0.23			
E1 (vs. E0)	2.47	1.08–5.67	0.032	1.118	0.470–3.004	0.7164
S1 (vs. S0)	1.97	0.86–4.50	0.109			
T1-2 (vs. T0)	3.39	1.50–7.65	0.003	2.426	1.053–5.590	0.0374
C1-2 (vs. C0)	4.18	1.84–9.52	0.0006	4.062	1.709–9.656	0.0015
C3 >1+ (vs. ≤ 1+)	2.72	1.16–6.35	0.021	2.596	1.093–6.615	0.0306
Rejection (vs. no rej)^c^	2.28	0.92–5.62	0.074	1.267	0.461–3.480	0.6459
DD (vs. LR + LU)	0.60	0.27–1.33	0.21	0.106	0.031–0.358	0.0003
DD (vs. LR)	0.56	0.24–1.27	0.17	0.115	0.034–0.390	0.0005
Months transplant to biopsy	1.004^b^	0.99–5.62	0.18			

CI, confidence interval; DD, deceased donor; LR, living related donor; LU, living unrelated donor.

For the 3-parameter multivariable analyses, serum creatinine (SCr) and urine protein/creatinine ratio (Up/c) remained significant in all cases with *P*-values < 0.001 and hazard ratios of 1.879 (SCr) and 1.389 (Up/c) with E1, 1.896 (SCr) and 1.362 (Up/c) with T1-2, 1.969 (SCr) and 1.474 (Up/c) with C1-2, 3.229 (SCr) and 1.736 (Up/c) with C3 >1+, 1.876 (SCr) and 1.387 (Up/c) with rejection, 2.575 (SCr) and 1.666 (Up/c) with DD versus LR + LU, 2.701 (SCr) and 1.667 (Up/c) with DD versus LR.

Biopsies with donor-derived IgA deposits are not included. Of the 72 biopsies with cell-mediated rejection 29 of 72 (40%) had C3 >1+; of those performed for proteinuria 24/52 (46%) had C3>1+.

a By Cox model.

b Per 1 unit increase for serum creatinine and urine protein/creatinine ratio, and per 1 month increase for months transplant to biopsy.

c Cell-mediated rejection, Banff grade 1A or higher.

## Discussion

In our experience, the majority of kidney transplant biopsies performed beyond the first 2 months posttransplantation that show glomerular IgA deposits represent recurrent IgAN, although a significant minority represent *de novo* IgAN, which is likely to have a different clinical implication than recurrent disease. Although both recurrent and *de novo* IgAN may be seen both early and late posttransplantation with a mean time of diagnosis of approximately 7.5 years posttransplantation and a similar frequency of segmental glomerulosclerosis and interstitial fibrosis/tubular atrophy, recurrent disease is more often associated with active glomerular inflammation (mesangial and endocapillary hypercellularity and crescents) and greater glomerular C3 deposition. At the other end of the pathologic spectrum, glomerular IgA deposits of donor origin were not found to be associated with either active or chronic histologic lesions or more than very mild glomerular C3 staining. The absence of glomerular lesions in such biopsies is consistent with the findings of a report of 164 donor kidney biopsies with glomerular IgA, which showed mesangial hypercellularity and segmental glomerulosclerosis in just 8% and 3%, respectively; although in this study, glomerular C3 staining of unspecified intensity was seen in the majority of cases.^22^ Notably, there was no significant impact of donor IgA on graft survival over a 12-year interval.

In 27% (72/264) of our kidney transplant biopsies with glomerular IgA deposits, the patient’s native kidney disease was not known. Because recurrent and *de novo* IgAN are likely to have different clinical implications, the pathologic findings in cases where the patient’s native kidney disease is not known may be of some help in assessing whether the IgA deposits present are more likely to represent recurrent or *de novo* disease. Specifically, biopsies showing active glomerular inflammatory lesions (M1, E1, and/or C1) and > 1+ C3 staining are more likely to represent recurrent disease and are probably best treated as such.

The significant association of higher SCr levels; proteinuria; Oxford E, T, and C scores; and intensity of glomerular C3 staining in recurrent IgAN (and in combined recurrent and *de novo* IgAN) with reduced graft survival is highly consistent with the impact of each of these parameters on native kidney survival^2^^,^^14^, ^15^, ^16^^,^^23^^,^^24^ and is consistent with previous reports in kidney allografts.^6^^,^^8^^,^^9^^,^^12^ Furthermore, in cases of recurrent IgAN, the Oxford T and C scores remained significant predictors of graft loss independent of SCr and Up/c levels although the E score did not. Interpretation of the significance of endocapillary hypercellularity in allografts with IgAN is difficult because this cannot be distinguished from glomerulitis, which is a manifestation of AMR.^18^ For this reason we specifically excluded biopsies diagnostic for AMR from this study; however, there are incomplete forms of AMR (e.g., microvascular inflammation, C4d- and donor-specific antibody negative) that also impact graft survival although to a lesser extent than full-blown AMR.^18^^,^^25^ Still, these findings stress the importance of routinely performing immunofluorescence (or immunohistochemical) studies for Igs and complement components on kidney allograft biopsies performed for allograft dysfunction and/or proteinuria, correlating the results of these findings with information from the native kidney whenever possible, quantitating proteinuria in these patients, and scoring all kidney transplant biopsies with IgAN using the Oxford MEST-C scores.^21^ Although the most recently published Kidney Disease Improving Global Outcomes guidelines^26^ do not recommend using the MEST-C scores as the basis for guiding therapy in patients with native kidney IgAN; this may be subject to change with recent emergence of many new drugs for treating patients with IgAN that have now been approved or are in clinical trials.^13^^,^^27^^,^^28^ Inclusion of these histologic scores are a component of the IgAN risk prediction tool,^29^ the use of which is recommended by Kidney Disease: Improving Global Outcomes,^26^ and allow for earlier risk stratification of patients with IgAN than clinical parameters alone. Furthermore, recent practice recommendations for treating IgAN in pediatric patients stress the importance of performing an early kidney biopsy and considering the use of immunosuppressive therapy in patients at clinical risk of progression, the latter including M1, E1, and C1 lesions.^30^ The similarity of impact of these histologic scores as well as of proteinuria on clinical outcomes in native kidney and recurrent IgAN suggests that these recommendations for native kidney IgAN are highly likely to be applicable to recurrent disease as well. It should also be considered that active glomerular lesions of IgAN in the allograft are likely to represent particularly severe disease, given that these patients are already on strong immunosuppressive medications to prevent rejection.

An interesting and somewhat unexpected finding is that recurrent IgAN occurring in living related allografts had reduced postbiopsy graft survival compared with lesions occurring in deceased-donor grafts when adjusting for SCr and Up/c at the time of biopsy. Genetic factors impact both susceptibility to and severity of IgAN in native kidneys,^31^ and some but not all studies have shown a higher recurrence rate of IgAN in kidneys from related versus unrelated donors.^32^, ^33^, ^34^ However, it is not generally advised to avoid living related donors for potential transplant recipients with IgAN because of the graft survival advantages of living donor versus deceased donor organs^35^ and the fact that IgAN, when it recurs, tends to occur rather late during the posttransplant period.^36^ However, our findings in this study, albeit in a limited patient sample, suggest that in addition to a potentially higher risk for recurrence of IgAN, patients with recurrent IgAN in living related allografts have a greater risk of graft loss than those with recurrent disease in deceased-donor grafts. Perhaps the genetic factors that help drive glomerular inflammation in IgAN become even more crucial in the transplant setting when this inflammation must overcome baseline immunosuppression. It also cannot be excluded that the deceased donor subgroup may have included a small number of patients with donor-derived IgAN (which is associated with good outcomes^22^) because deceased donors are less likely than live donors to be screened for urinary abnormalities, although the fraction of such patients would be expected to be small. Although this finding requires validation in larger and multicenter studies, it does provide a warning against the possible use of related donors in potential transplant recipients with IgAN, especially in the future when antirejection regimens hopefully continue to be improved and graft loss due to rejection during the first 5 or even 10 years posttransplantation becomes less common.

This study has several important limitations. It is a single-center retrospective study, only a minority of the patients with recurrent and *de novo* IgAN had available follow-up data, and the mean postbiopsy follow-up in these patients who did not develop graft loss was not particularly long, just over 5 years. Furthermore, in approximately one-fourth of the patients with glomerular IgA deposits in their allograft biopsy, their native kidney disease was not known; it is felt that this is related to the majority of our biopsies being received from > 30 outside hospitals and laboratories that provide variable amounts of accompanying clinical information. Therefore, the number of cases included in the analyses of graft survival was relatively small, especially for *de novo* IgAN. There were also no apparent demographic features (patient age and sex, time posttransplantation of biopsy) that distinguished recurrent from *de novo* IgAN, although the fractions of biopsies with glomerular IgA deposits and unknown native kidney disease that showed rejection and that were performed for proteinuria (Table 1) suggest that most of these biopsies had *de novo* rather than recurrent IgAN. Still, the latter appears to be considerably more common than *de novo* IgAN, although because the patients in our study did not have protocol biopsies, it is not possible to estimate the frequency with which IgAN recurs in kidney allografts. The lack of protocol biopsies prevented us from determining the impact of recurrent IgAN on graft survival by comparing outcomes in these patients with those in patients with native kidney IgAN and documented absence of glomerular IgA deposits in their allografts, although a previous study^36^ of transplant recipients with native kidney IgAN suggested that graft survival curves of patients with and without recurrence are initially similar and begin to deviate with an increased rate of graft loss in the recurrent IgAN cohort after 6 to 7 years posttransplantation, which is just slightly less than the mean posttransplant interval for diagnosis of recurrent IgAN in our study (91 months). Finally, as noted above, the true impact of endocapillary hypercellularity on the outcomes of IgAN in transplanted kidneys could not be accurately determined because of the overlap of this lesion with glomerulitis. Still, our findings confirm that recurrent IgAN is clearly not a benign condition^37^ and not infrequently leads to graft loss. Clinical and histological parameters associated with reduced kidney survival in native kidney IgAN, including reduced kidney function, proteinuria, Oxford MEST-C scores (particularly T and C scores), and glomerular complement activation as assessed by C3 staining, are also quite relevant to predicting the likelihood of graft loss in patients with recurrent IgAN. Finally, recurrent IgAN should be distinguished from *de novo* IgAN and donor-derived mesangial IgA deposits, which are associated with less glomerular inflammation and a more benign clinical course.

## Disclosure

MH serves on the adjudication committee for an industry-sponsored clinical trial (AstraZeneca – Treatment of Proliferative Lupus Nephritis) and has also received honoraria for serving as an advisor for Novartis, Argenx, Otsuka, Biogen, Travere and Vera Therapeutics. All the other authors declared no competing interests.

## Supplementary Material

Supplementary File (PDF) **Table S1.** Comparison of recurrent and *de novo* IgA nephropathy in allografts with follow-up. **Table S2.** Clinical and pathologic parameters associated with graft loss in recurrent + *de novo* IgA nephropathy. STROBE statement.

## Footnotes

Supplementary File (PDF)
**Table S1.** Comparison of recurrent and *de novo* IgA nephropathy in allografts with follow-up. **Table S2.** Clinical and pathologic parameters associated with graft loss in recurrent + *de novo* IgA nephropathy. STROBE statement.
