# Supplementary material for: Kidney Transplant Outcomes in Recurrent Versus De Novo IgA Nephropathy
Source: Kidney Int Rep. 2025 May 21;10(8):2659–67. doi: 10.1016/j.ekir.2025.05.014 (PMC12348186; doi:10.1016/j.ekir.2025.05.014)
Supplement: Supplementary File (PDF) — Table S1. Comparison of recurrent and de novo IgA nephropathy in allografts with follow-up. Table S2. Clinical and pathologic parameters associated with graft loss in recurrent + de novo IgA nephropathy. STROBE statement. [file mmc1.pdf]

Table S1. Comparison of Recurrent and De Novo IgA Nephropathy in Allografts with Follow-Up

	Recurrent (54)		De Novo (20)	
Graft Loss	Yes	No	Yes	No
Number (%)	25 (46)	29 (54)	5 (25)	15 (75)
Post-bx Follow-up, mo				
Mean	32	63	42	61
SD	28	37	15	46
Range	2 - 109	15 - 156	19 - 56	10 - 175
Sex M/F	17/8	21/8	3/2	12/3
Donor Type				
Deceased	14	20	3	11
Living Rel.	10	7	1	4
Living Unrel.	1	2	1	0
Age at Biopsy, y, mean	50	46	35	47
SD	13	13	15	15
Range	26 - 78	16 - 76	19 - 63	21 - 67
Biopsy Months Post-Tx				
Mean	100	79	113	90
SD	61	66	76	70
Range	3 - 248	3 - 264	6 - 210	4-234

Serum Creatinine (mg/dL)				
Mean	3.1	1.7	2.3	1.7
SD	1.3	0.7	0.3	0.6
Range	1.8 - 7.9	0.5 - 4.1	1.7 - 2.6	0.5 - 3.4
Urine p/c				
Mean	2.8	1.1	1.1	1.0
SD	1.9	1.5	1.1	0.9
Range	0.2 - 7.6	0.1 - 8.0	0.1 - 2.5	0.1 - 3.0
U p/c $\geq$1, no. (%)	21 (84)	11 (38)	2 (40)	5 (33)
Pathologic Findings				
M1, no. (%)	14 (56)	13 (45)	1 (20)	4 (27)
E1, no. (%)	10 (40)	5 (17)	1 (20)	1 (7)
S1, no. (%)	15 (60)	14 (48)	1 (20)	3 (20)
T1-2, no. (%)	15 (60)	7 (24)	2 (40)	4 (27)
C1-2, no. (%)	11 (44)	1 (3)	0	0
C3 >1+, no. (%)	17 (68)	11 (38)	1 (20)	3 (20)
Rej., no. (%)	6 (24)	5 (17)	2 (40)	2 (13)

Table S2. Clinical and Pathologic Parameters Associated with Graft Loss in Recurrent + De Novo IgA Nephropathy

	Univariate			With SCr and Up/c		
Parameter	Hazard ratio[1]	95% CI	P value[2]	Hazard Ratio[1]	95% CI	P value[2]
Serum creatinine (SCr, mg/dL)	1.97	1.52 - 2.55	<0.0001			
Urine prot/creat ratio (Up/c, g/g)	1.49	1.27 - 1.75	<0.0001			
M1 (versus M0)	1.80	0.87 - 3.72	0.11			
E1 (versus E0)	3.28	1.52 – 7.10	0.0003	1.506	0.618 - 3.666	0.3676
S1 (versus S0)	1.97	0.98 - 4.29	0.057			
T1-2 (versus T0)	3.26	1.57 – 6.79	0.002	2.113	0.997 – 4.475	0.0508
C1-2 (versus C0)	5.06	2.33 – 10.98	<0.0001	4.068	1.816 - 9.113	0.0006
C3 >1+ (versus ≤1+)	2.88	1.38 - 6.05	0.005	2.378	1.120 – 5.048	0.0241
Rejection (versus no rej)[3]	2.28	1.04 - 5.07	0.040	1.632	0.684 – 3.891	0.2692
DD (versus LR + LU)	0.64	0.31 - 1.31	0.22	0.173	0.064 - 0.464	0.0005
DD (versus LR)	0.61	0.28 - 1.30	0.20	0.182	0.067 - 0.496	0.0009
Months transplant to biopsy	1.005	0.99 – 1.01	0.086			

Legend to Table S2

[1]Per 1 unit increase for serum creatinine and urine protein/creatinine ratio, and per 1 month increase for months transplant to biopsy

[2]By Cox model

[3]Cell-mediated rejection, Banff grade 1A or greater

For the 3-parameter multivariable analyses, serum creatinine (SCr) and urine protein/creatinine ratio (Up/c) remained significant in all cases with P values <0.001 and hazard ratios of 1.898 (SCr) and 1.386 (Up/c) with E1, 1.917 (SCr) and 1.390 (Up/c) with T1-2, 1.933 (SCr) and 1.464 (Up/c) with C1-2, 1.911 (SCr) and 1.446 (Up/c) with C3 >1+, 2.575 (SCr), 1.913 (SCr) and 1.399 (Up/c) with rejection, 2.515 (SCr) and 1.619 (Up/c) with DD versus LR + LU, 2.463 (SCr) and 1.619 (Up/c) with DD versus LR. DD = deceased donor; LR = living related donor, LU = living unrelated donor, CI = confidence interval

STROBE Statement—checklist of items that should be included in reports of observational studies

	Item No.	Recommendation	Page No.	Relevant text from manuscript
**Title and abstract**	1	(*a*) Indicate the study's design with a commonly used term in the title or the abstract	2	Abstract line 4
		(*b*) Provide in the abstract an informative and balanced summary of what was done and what was found	2	Abstract lines 4-7; Abstract lines 8 -16
**Introduction**				
Background/rationale	2	Explain the scientific background and rationale for the investigation being reported	4	par.. 1, lines 4-7, par. 2 lines 1-2
Objectives	3	State specific objectives, including any prespecified hypotheses	5	lines 7-14
**Methods**				
Study design	4	Present key elements of study design early in the paper	6	par. 1 lines 1-5, 12-20
Setting	5	Describe the setting, locations, and relevant dates, including periods of recruitment, re-exposure, follow-up, and data collection	6-7	p. 6, par. 1, lines 1-5, 14-22<br>p. 7, lines 1-11
	6	(*a*) *Cohort study*—Give the eligibility criteria, and the sources and methods of selection of participants. Describe methods of follow-up<br>*Case-control study*—Give the eligibility criteria, and the sources and methods of case ascertainment and control selection. Give the rationale for the choice of cases and controls<br>*Cross-sectional study*—Give the eligibility criteria, and the sources and methods of selection of participants	6-7	p. 6, par. 1 lines 1-5, 7-10<br>p. 7 par. 1 lines 5-10, par. 2 lines 3-5
		(*b*) *Cohort study*—For matched studies, give matching criteria and number of exposed and unexposed<br>*Case-control study*—For matched studies, give matching criteria and the number of controls per case	8,9	p. 8, par. 1 lines 6-11<br>p. 9, par. 3, lines 1-3
Variables	7	Clearly define all outcomes, exposures, predictors, potential confounders, and effect modifiers. Give diagnostic criteria, if applicable	7-8	p 7, par. 2, lines 1-5<br>p. 8, par. 1, lines 1-11

Data sources/ measurement	8*	For each variable of interest, give sources of data and details of methods of assessment (measurement). Describe comparability of assessment methods if there is more than one	6-7	p. 6, par. 1, lines 12-22<br>p. 7, par. 1 lines 1-11
Bias	9	Describe any efforts to address potential sources of bias	6,8,10	p. 6, par. 1, lines 10-12; p. 8 par. 3, lines 1-3, p. 10 lines 8-9
Study size	10	Explain how the study size was arrived at	8,9	p. 8, par. 1, lines 1-3, p. 9, par. 3, line 1

n

Quantitative variables	11	Explain how quantitative variables were handled in the analyses. If applicable, describe which groupings were chosen and why	7	par.2, lines 1-3
Statistical methods	12	(*a*) Describe all statistical methods, including those used to control for confounding	7	par. 2, lines 3-5
		(*b*) Describe any methods used to examine subgroups and interactions	7	par. 2, lines 3-5
		(*c*) Explain how missing data were addressed	n/a	
		(*d*) *Cohort study*—If applicable, explain how loss to follow-up was addressed<br>*Case-control study*—If applicable, explain how matching of cases and controls was addressed<br>*Cross-sectional study*—If applicable, describe analytical methods taking account of sampling strategy	20,21	Fig 1 legend, lines 1-2<br>Fig 2 legend lines 1-2
		(*e*) Describe any sensitivity analyses		
**Results**				
Participants	13*	(a) Report numbers of individuals at each stage of study—eg numbers potentially eligible, examined for eligibility, confirmed eligible, included in the study, completing follow-up, and analysed	8, 9	p. 8, par. 1; p. 9, par.
		(b) Give reasons for non-participation at each stage	n/a	
		(c) Consider use of a flow diagram	n/a	
Descriptive data	14*	(a) Give characteristics of study participants (eg demographic, clinical, social) and information on exposures and potential confounders	8, 9, Table 1	p. 8, par. 1
		(b) Indicate number of participants with missing data for each variable of interest	n/a	

		(c) *Cohort study*—Summarise follow-up time (eg, average and total amount)	Tables 1, S1	
Outcome data	15*	*Cohort study*—Report numbers of outcome events or summary measures over time	Figs 1,2;Table S1	
		Case-control study—Report numbers in each exposure category, or summary measures of exposure		
		Cross-sectional study—Report numbers of outcome events or summary measures		
Main results	16	(*a*) Give unadjusted estimates and, if applicable, confounder-adjusted estimates and their precision (eg, 95% confidence interval). Make clear which confounders were adjusted for and why they were included	9,10; Tables 3 and S1	p. 9, bottom par., an
		(*b*) Report category boundaries when continuous variables were categorized	Table S1	
		(*c*) If relevant, consider translating estimates of relative risk into absolute risk for a meaningful time period	n/a	

Continued on next page

Other analyses	17	Report other analyses done—eg analyses of subgroups and interactions, and sensitivity analyses 10,	10, Tables 3 and S2	p. 10, lines 8-12
**Discussion**				
Key results	18	Summarise key results with reference to study objectives	11,12	p. 11, par. 1, lines 1- 11; lines 1-7
Limitations	19	Discuss limitations of the study, taking into account sources of potential bias or imprecision. Discuss both direction and magnitude of any potential bias	12,14	p. 12, lines 7-12; p. 14 lines 1-10
Interpretation	20	Give a cautious overall interpretation of results considering objectives, limitations, multiplicity of analyses, results from similar studies, and other relevant evidence	13,14	p. 13, last 8 lines, and p.
	21	Discuss the generalisability (external validity) of the study results	11,12,13	p. 11, par. 1, last 6 lines lines 1-5; p. 13, lines 2-7
**Other information**				
Funding	22	Give the source of funding and the role of the funders for the present study and, if applicable, for the original study on which the present article is based	15	Disclosures lines 1-2

*Give information separately for cases and controls in case-control studies and, if applicable, for exposed and unexposed groups in cohort and cross-sectional studies.

Note: An Explanation and Elaboration article discusses each checklist item and gives methodological background and published examples of transparent reporting. The STROBE checklist is best used in conjunction with this article (freely available on the Web sites of PLoS Medicine at http://www.plosmedicine.org/, Annals of Internal Medicine at http://www.annals.org/, and Epidemiology at http://www.epidem.com/). Information on the STROBE Initiative is available at www.strobe-statement.org.
